# A commentary on ‘High ligation of the anal fistula tract by lateral approach: A prospective cohort study on a modification of the ligation of the intersphincteric fistula tract technique’

**DOI:** 10.1097/JS9.0000000000001270

**Published:** 2024-03-04

**Authors:** Shiwei Yang, Liwei Yan, Keliang Jia, Chao Gu, Guang Zhang

**Affiliations:** a Department of General Surgery, The First Affiliated Hospital of Shandong First Medical University; b Department of Health Management, The First Affiliated Hospital of Shandong First Medical University; c Department of Anorectal Surgery, Affiliated Hospital of Shandong University of Traditional Chinese Medicine, Jinan, Shandong, People’s Republic of China

*Dear Editor*,

The ligation of the intersphincteric fistula tract (LIFT) technique mainly ligates and cuts the fistula between the sphincters, scrapes the infected tissue of the fistula wall, and tightens the fistula tract with ligation, which is an effective and low-cost sphincter-sparing technique with a success rate of 94.4% and the absence of continence failures^1,2^. Kang *et al*.^3^ conducted a prospective analysis on patients who were scheduled for high ligation of the fistula tract using the lateral route. For transsphincteric anal fistulas, high ligation of the anal fistula tract via lateral approach may be a helpful sphincter-sparing treatment.

Between December 2015 and July 2017, 28 patients participated in this prospective trial. The follow-up period ranged from 8 to 27 months, with a median of 16 months. Nine patients (32%) had complex transsphincteric fistulas, whereas the remaining 19 patients (68%) had simple ones. According to the study’s findings, successful fistula closure occurred in 21 patients (75%), with a median healing period of 4 weeks (range, 3–7 weeks). After the operation, none of the patients reported any incontinence issues. Furthermore, two (7%) of the seven patients (25%) who did not heal successfully did not recover up to 2 months after surgery, and five (18%) experienced recurrence after complete healing.

The study also reveals several shortcomings of the lateral approach strategy when compared to the original LIFT technique. First, ligation can be achieved through the intersphincteric incision in the original LIFT technique, even in cases where the external fistula tract is extremely long and curved. However, using the lateral approach technique to lead to the intersphincteric space by coring from the external opening presents some technical challenges. Second, because of ‘downstaging,’ if a transsphincteric fistula recurs after the first LIFT procedure, a new external opening may occasionally form on the site of the newly formed intersphincteric incision rather than the initial external opening site. This makes the subsequent procedure considerably simpler. But there remained a great deal of limitations. First, it’s possible that certain residual confounding variables were overlooked. A bad prognosis is linked to risk variables such as sex, fistula type, and location. Second, the bias exists since there is no control group. Finally, the patient’s history, including comprehensive personal history such as diabetes, hypertension, or comorbidities, should be documented, as this will influence wound healing^4^.

This study delves into confirming the usefulness of this sphincter-saving procedure. This issue has enormous ramifications for clinical practice. This forward-thinking approach provides valuable guidance and inspiration for the continued development of this area of research. Due to the characteristics of multiple inducements and forms of complex anal fistula, there is a lack of unified criteria for the efficacy evaluation of various anal fistula treatment methods, and no consensus has been reached on the best therapy. According to current research findings, several combined techniques based on drainage Seton [pulling Seton, external anal sphincter (EAS)-sparing Seton after rerouting, and rerouting Seton around the EAS combined with mucosal advancement flap] and LIFT-plug are considered feasible therapies for complex anal fistulas, but their clinical outcomes require more prospective randomized controlled trials with multicenter, large sample size, and long-term follow-up^5^. In the future, a significant number of high-quality clinical trials are urgently needed to jointly resolve the question of the optimum method for anal fistula treatment.

## Ethical approval

This manuscript is a comment. Don’t need ethical approval.

## Consent

This manuscript is a comment. Don’t need patient consent.

## Author contribution

S.Y. and L.Y.: study concept or design, data collection, data analysis or interpretation, and writing the paper; K.J. and C.G.: data collection; G.Z.: study concept or design, writing, and revising the paper.

## Conflicts of interest disclosure

This manuscript is a comment without conflicts of interest.

## Research registration unique identifying number (UIN)

This manuscript is a comment. Don’t need UIN.

## Guarantor

Guang Zhang.

## Data availability statement

This manuscript is a comment. Don’t need a Data availability statement. However, all the data from the current study are publicly available.

## Provenance and peer review

This manuscript is a comment without being invited.
